# Multidisciplinary collaboration in diagnosis and treatment of neuroleptic malignant syndrome complicated with pneumonia: report of three cases

**DOI:** 10.1186/s13000-022-01270-z

**Published:** 2022-11-09

**Authors:** Huachang Zhao, Sijia Lu, Xiaoyan Li, Panyun Huang, Hongming Wang, Fengmei Lu

**Affiliations:** 1 The Clinical Hospital of Chengdu Brain Science Institute, University of Electronic Science and Technology of China, Chengdu, 611731 China; 2 The Second Internal Medicine Department, the Fourth People’s Hospital of Chengdu, Chengdu, 610036 China

**Keywords:** Neuroleptic malignant syndrome, Pneumonia, Respiratory failure, Antipsychotics, Psychiatric

## Abstract

**Background:**

Neuroleptic malignant syndrome (NMS) is a relatively rare and a potentially fatal syndrome. It is a serious complication associated with antipsychotic therapy. NMS is easily prone to pneumonia, rhabdomyolysis and other problems. However, the clinical features of NMS complicated with pneumonia remains largely unclear.

**Case presentation:**

Here, we described three female adult patients of NMS complicated with pneumonia in our own hospital. The symptoms of the patients were controlled with antipsychotic drugs at admission. Symptoms such as high fever, high muscle tone, difficulty in eating, phlegm in the throat, anhelation, rhabdomyolysis and autonomic nervous dysfunction occurred 2 days after the treatment, which mainly concentrated within 1 week. In addition, they are all healed.

**Conclusions:**

NMS is a rare and serious complication in psychiatric department, which is easy to be complicated with pneumonia and respiratory failure. Timely identification and early intervention could help achieve a good prognosis.

## Introduction

Neuroleptic malignant syndrome (NMS) is a relatively rare and a potentially fatal syndrome that occurs in association with the use of neuroleptic drugs [1]. It was first described in 1960 by the French clinicians Delay and colleagues, which they called “akinetic hypertonic syndrome” [2]. NMS is a neurological emergency characterized by hyperthermia, elevated creatine kinase, altered mental status, and autonomic nervous instability [3]. Due to its scattered visits and insufficient understanding of non-professional physicians, it is easy to cause missed diagnosis and misdiagnosis, and its potential mortality is high [4], which should be paid close attention to by clinicians, especially physicians. After 60 years of development, however, currently there are still few systematic studies on the NMS.

NMS complicated with pneumonia increases the difficulty of clinical treatment. We have encountered three cases of NMS complicated with pneumonia during our clinical work. In this paper, multidisciplinary collaboration in diagnosis and treatment of 3 cases of NMS complicated with pneumonia is presented.

## Case presentations

### Case 1

Case 1 is a 52-years-old married and unemployed female. She was admitted to the hospital on January 27, 2020 due to “nervousness, self-talk, abnormal speech and behavior for 13 years and getting worse for 3 days”. Thirteen years ago, she presented with nervousness, fear, self-talk, guilt, abnormal behavior, and inability to sleep, without any inducements. She was diagnosed with “schizophrenia” and treated with clozapine and sulpiride by the local hospital. Because of leukopenia, drug therapy was changed to “risperidone 2 mg qd and olanzapine 10 mg qn” long-term treatment, then the condition was well controlled, and daily life was self-care. In the past year, she reduced the medication due to the improvement of symptoms. Three days ago, she was admitted to the psychiatric department in our hospital due to that the above symptoms aggravated and she cut her wrist. Admission examinations revealed the following: body temperature (T), 36.5℃, respiratory rate (R), 18 times/min, pulse (P), 85 beats/min, and blood pressure (BP), 110/70 mmHg. Symptoms included poor general condition, stiff and speechless, dull expression, air pillow and wax flexion, active violation, left wrist injury, other physical examinations and routine biochemical examinations showed no abnormalities. Admission diagnosis was schizophrenia and stupor, and then she received haloperidol injection 10 mg im bid, nutritional support treatment, and indwelling gastric tube. On the second day after admission, the patients developed fever (T 37.3℃), and R was 30 times/min, P was 156 beats/min, BP was 140/97 mmHg, and muscle tone +  +  +  + . She also presented with profuse sweating, tremor, phlegm in the throat, gastric retention, and urinary retention. Haematological investigations were as follows: white blood cell (WBC), 6.89 × 10^9^/l, neutrophil (N), 80.1%, C-reactive protein (CRP), 69.72 mg/l, serum amyloid a (SAA), > 200 mg/l, and procalcitonin (PCT), 0.678 ng/ml. Lung computer tomography (CT) scan result showed small pieces of fuzzy shadows scattered in the middle and lateral lobes of the right lung.

Considering the schizophrenia complicated by pneumonia, she was started on “risperidone 2.5 mg qd, lorazepam 0.5 mg tid, cefoperazone 2 g ivgtt q8h, as well as routine expectorant treatment, airway management, and nutritional support” treatment. On the 4th day of admission, she presented with persistent fever (T 39.5℃), R was 35 ~ 60 times/min, P was 120 ~ 160 times/min, BP was 130 ~ 170/80-110 mmHg. She also demonstrated irritability and lethargy alternately, phlegm in the throat, no active cough, and right lung rale. Arterial blood gas analysis showed type I respiratory failure and decompensated respiratory alkalosis. Blood routine tests results indicated the following: WBC, 14.02 × 10^9^/l, N, 89.2%, CRP, 125 mg/l, SAA, > 200 mg/l, PCT, 1.043 ng/ml, myohemoglobin (Myog), > 1000 ng/ml, creatine kinase (CK), 1170 U/L, D dimer (DD), 2.54 ug/ml, fibrinogen (FDP), 5.67 ug/ml, and lactic acid (Lac), 1.0 mmol/l. Sputum culture result showed Staphylococcus aureus and cefoperazone sensitive to sulbactam. Electrocardiogram (ECG) result revealed sinus tachycardia. Then she was transferred to respiratory department, after multidisciplinary (MDT) discussion, she was diagnosed with NMS, severe pneumonia, schizophrenia and secondary myolysis. The antipsychotic drugs were stopped, and propranolol 10 mg tid, lorazepam 1 mg q12h were used. In addition, the patient received hydration therapy to maintain urine output ≥ 2000 ml/d and anti-infection expectorant regimen continued with non-invasive ventilator support. On the 6th day of admission, her body temperature was 38.5℃, R was 35^±^ times/min, P was 120^±^ beats/min, BP was 145/90 mmHg, and her symptoms such as sweating and phlegm in the throat were relieved. Blood gas analysis suggested compensatory respiratory alkalosis.

Continue with the original treatment plan, on the 9th day of admission, she presented with T 38℃, R 28^±^ times/min, P 100^±^ beats/min, BP 148/89 mmHg, no visible sweat on the skin, no tremor, muscle tone +  + , and the symptoms of phlegm in the throat and rales in both lungs disappeared. Blood routine tests demonstrated the following: WBC, 7.62 × 10^9^/l, N, 79.2%, CRP, 14.65 mg/l, SAA, 58.12 mg/l, PCT, 0.1 ng/ml, Myog, 26.296 ng/ml, CK, 348 U/L, and no other abnormalities. Lung CT scan showed that the original lung patch shadow was completely absorbed. On the 14th day of admission, she presented a temperature of 36.8℃ and routine tests as well as biochemical examinations were normal. Cefoperazone was stopped and hydration treatment was performed. Due to obvious mental symptoms, her family members strongly requested to use the original risperidone treatment regimen. Based on the psychiatrist evaluation, she was started on 1 mg qn, and she got fever again (T 38.9℃) on the 16th day with R 40^±^ times/min, P 130^±^ beats/min, BP 140/100^±^mmHg, muscle tone +  +  +  + , and also presented with profuse sweating, tremor, and phlegm in the throat. Blood routine tests results showed WBC, 7.17 × 109/l, N, 81.2%, CRP, 25.4 mg/l, SAA, 50 mg/l, PCT, 0.1 ng/ml, Myog, 300 ng/ml, CK, 879 U/L, and Lac, 2.4 mmol/l. Arterial blood gas analysis were decompensated respiratory alkalosis and metabolic acidosis. Then the risperidone was immediately stopped and she was given hydration, propranolol 20 mg tid, lorazepam 1 mg q12H, and scopolamine hydrobromide 0.3 mg im bid treatments. On the 20th day, her body temperature, respiratory pulse, sweating, and muscle tone were recovered. In order to control the psychiatric symptoms, after evaluation by MDT, she was received with amisulpride 0.2 g bid, lorazepam 1 mg qn, propranolol 10 mg tid, and trihexyphenidol 2 mg bid and then she was discharged. The detailed information about the laboratory tests was presented in Table 1.

**Table 1:** Changes in the laboratory tests in different days of Case 1

**Days after Admission**	**T (℃)**	**R(times/min)**	**P(beats/min)**	**BP (mmHg)**	**WBC (× 10**^**9**^**/l)**	**N (%)**	**CRP (mg/l)**	**SAA (mg/l)**	**PCT (mg/l)**	**Myog (ng/ml)**	**CK (U/L)**
0	36.5	18	85	110/70							
2	37.3	30	156	140/97	6.89	80.1	69.72	> 200	0.678		
4	39.5	35–60	120–160	130–170/80–110	14.02	89.2	125	> 200	1.043	> 1000	1170
6	38.5	35	120	145/90							
9	38	28	100	148/89	7.62	79.2	14.65	58.12	0.1	26.296	348
16	38.9	40	130	140/100	7.17	81.2	25.4	50	0.1	300	879

*Abbreviations*: *T* Body temperature, *R* Respiratory rate, *P* Pulse, *BP* Blood pressure, *WBC* White blood cell, *N* Neutrophil, *CRP* C-reactive protein, *SAA* Serum amyloid a, *PCT* Procalcitonin, *Myog* Myoglobin, *CK* Creatine kinase

### Case 2

Case 2 is a 49-years-old married and unemployed female. She was admitted to our hospital on February 14, 2020 due to “abnormal speech and behavior for 10 years and getting worse for 3 days”. Ten years ago, the patient ran away from home due to the death of her father. She was diagnosed as “mental retardation and schizophrenia” with speech disorder, emotional agitation, verbal abuse, and rambling during communication. The specific treatment was unknown and she stopped taking medicines due to the feeling of drowsiness after taking the medicine, and the above symptoms did not occur again. Three days ago, she was admitted to the psychiatric department in our hospital due to behaving disorderly and shouting to hack someone, etc. Admission examinations were as follows: T, 36.6℃, R, 20 times/min, P, 90 beats/min, BP, 130/84 mmHg, and no other special things. She was fully conscious, but uncooperative during physical examination, loose thinking, increased pathological will, lack of intelligence, muscle strength of level 5, muscle tone +  + , and no abnormalities in auxiliary examination. The admission diagnosis were mental retardation and acute schizophrenia-like psychotic disorder. Then she was received haloperidol injection 5 mg im bid, diazepam 10 mg qn with psychiatric monitoring and related assessment, and then her mental symptoms were improved. On the 3rd day of admission, she presented with T, 38.3℃, R, 20 times/min, P, 105 beats/min, BP, 128/76 mmHg, oxyhemoglobin saturation (SPO_2_), 98%. She was conscious, could normal communicate, and the breath sounds in both lungs were thick, had a little hump in the right lower lung and no other abnormalities. ECG results showed sinus tachycardia. In consideration with pneumonia, the patient was then treated with oral cefprozil 0.5 g bid.

On the 5th day of admission, the laboratory tests showed the following: T, 38.9℃, R, 30 times/min, P, 120 beats/min, BP, 125/90 mmHg, and SPO_2_, 97%. Additionally, she presented with coma, pupil diameter 2 mm, dull light reflection, moist skin, tremor, muscle tone +  +  +  + , and a little rales in the lower right lung and the pathological signs were not elicited. Blood routine tests results included WBC 15.3 × 10^9^/l, N, 84.6%, CRP, 46.38 mg/l, SAA, 61.99 mg/l, PCT, 0.84 ng/ml, and Lac, 1.1 mmol/l. Biochemical examinations showed that urea nitrogen (BUN) was 12.2 mmol/l, uric acid (UN) was 448 umol/l, glucose (GLU) was 12.33 mmol/l, and albumin (ALB) was 31.5 g/l. CT scan results indicated no obvious abnormalities in the head, and there were scattered patches in the posterior basal segment of the lower lobe in the right lung. Then the treatment was adjusted as cefoperazone tazobactam 2 g ivgtt q12h, and symptomatic supportive treatment and the psychiatric treatments continued. On the 8th day of admission, she developed continuous high fever, phlegm in the throat and then she was treated with oxygen therapy 3 l/min. After that, T was 39.7℃, SPO_2_ was 88%, R was 36 times/min, P was 138 beats/min, and BP was 113/78 mmHg, and no improvement in consciousness. Then she was transferred to respiratory department. The biochemical examinations results showed that: CK > 12000 U/L, Myog > 1000 ng/ml, troponin I (CTnI), 0.1 ng/ml, sodium (NA^+^), 161.7 mmol/l, chlorine (CL-), 128.5 mmol/l, potassium (K +), 3.42 mmol/l, BUN, 15.8 mmol/l, alanine transaminase (ALT), 282 U/L, aspartate transaminase (AST), 753 U/L, UN, 616 umol/l, WBC, 18.46 × 10^9^/1, N, 86.6%, CRP, 56.1 mg/l, and PCT, 0.999 ng/ml. Arterial blood gas analysis revealed compensatory perchloric acidosis, PO_2_ 62 mmHg, and hypertonic dehydration, and no other abnormalities. After MDT, she was diagnosed with NMS, pneumonia, secondary myolysis, and mental retardation. Antipsychotics was stopped and she received propranolol 10 mg tid, lorazepam 0.5 mg tid, and hydration therapy to correct hypertonic dehydration and hypokalemia, reduced glutathione 1.8 g ivgtt qd, cefepime 2 g ivgtt q12h, and non-invasive ventilator support, etc.

On the 10th day of admission, she presented with muscle tone +  +  + , T, 37.2℃, R, 24 score, P, 100, BP, 125/78 mmHg, and SPO_2_, 95%. The sputum culture result was multi-drug resistant Staphylococcus aureus (MRAS). She also had cough, phlegm in the throat, and no changes in the little rales of the lower right lung. Blood routine included NA^+^ 146.2 mmol/l, WBC 12.58 × 10^9^/l, N 85.58%, CRP 41.09 mg/l, PCT 0.124 ng/ml. Arterial blood gas analysis showed mild metabolic acidosis. FDP was 7.48 ug/ml, D-D5.35 ug/ml, and she was treated with plus low molecular weight heparin 2500u ih q12h. On the 15th day of admission, she had a T, 37.6℃, R, 22 times/min, P, 84 beats/min, BP, 129/85 mmHg, SPO_2_, 95%, and clear consciousness, no dry or wet sounds in both lungs. She still had nervousness, tremor, sweating, and muscle tone +  + . On the 18th day of admission, the patient’s condition was further relieved, and she could communicate and T was 37.3℃, R was 21 times/min, and P was 87 beats/min. Examinations showed CK, 4500 U/L, Myog, 552.68 ng/ml, ALT, 200 U/L, AST, 206 U/L, and CT scan results showed absorption of lung lesions, and no other abnormalities. She self-reported that she had hallucinations and strong sense of victimization. After MDT consultation, it was considered that the malignant syndrome has been corrected and the pneumonia has been cured, mainly with psychiatric symptoms. The anti-infection regimen was stopped and she was treated with aripiprazole 5 mg qd. On the 21st day of admission, the condition deteriorated with fever (T 37.9℃), R was 30 times/min, P was 110 beats/min, and she had profuse sweating, fasciculation tremor, muscle tone +  +  + , and phlegm in the throat. Biochemical examinations showed that CK was 8000 U/L and Myog was 635.4 ng /ml. In considering the recurrence of NMS, aripiprazole was stopped and the hydration therapy, propranolol 10 mg tid, lorazepam 0.5 mg tid and other treatments were started. The patient’s condition was improved on the 23rd day of admission. After MDT discussion, we determined that the patient was not suitable for antipsychotic drugs. The non-convulsive electroconvulsive therapy (MECT) was performed on the patient after the muscle enzymes were normal. The detailed information about the laboratory tests was presented in Table 2.

**Table 2:** Changes in the laboratory tests in different days of Case 2

**Days after Admission**	**T (℃)**	**R(times/min)**	**P(beats/min)**	**BP (mmHg)**	**WBC (× 10**^**9**^**/l)**	**N (%)**	**CRP (mg/l)**	**PCT (mg/l)**	**Myog (ng/ml)**	**CK (U/L)**	**SPO**_**2**_** (%)**
0	36.6	20	90	130/84							
3	38.3	20	105	128/76							98
5	38.9	30	120	125/90	15.3	84.6	46.38	0.84	1.1		97
8	39.7	36	138	113/78	18.46	86.6	56.1	0.999	> 1000	> 12000	88
10	37.2	24	100	125/78			41.09	0.124			95
15	37.6	22	84	129/85							95
18	37.3	21	87						552.68	4500	
21	37.9	30	110						635.4	8000	

*Abbreviations*: T Body temperature, *R* Respiratory rate, *P* Pulse, *BP* Blood pressure, *WBC* White blood cell, *N* Neutrophil, *CRP* C-reactive protein, *PCT* Procalcitonin, *Myog* Myoglobin, *CK* Creatine kinase, *SPO*_*2*_ Oxyhemoglobin saturation

### Case 3

Case 3 is a 40-years-old married and unemployed female. She was admitted to our hospital at 18:07 on February 20, 2017 due to “self-talk and self-laugh for 10 years and getting worse for 2 weeks”. She has a family history of maternal schizophrenia. For 4 years, she talked to herself with messy content, laughed at herself, and scolded people without any inducements. She could take care of herself and did not receive any diagnosis and treatments. Her condition was exacerbated two weeks ago with insomnia, grabbing objects out of thin air, turning over trash cans, auditory hallucinations, and feeling victimized. She was considered as “schizophrenia-like disorder” and was given “olanzapine 20 mg qd” in the clinic. However, she refused to take medications, and she was admitted to acute psychiatric department since her condition aggravated. Physical examinations revealed the following: T, 36.5℃, R, 20 times/min, P, 80 beats/min, BP, 110/70 mmHg. The general condition was slightly worse, and she had clear consciousness, but uncooperative and uncoordinated emotional responses. There were no special abnormalities in the heart, lungs and abdomen. The diagnosis and treatment plan was haloperidol injection 5 mg im q12h, diazepam injection 10 mg qn, and psychiatric routine nursing. Others included the routine, biochemistry, and imaging examinations, psychopsychological assessment and monitoring of adverse drug reactions. The patient was temporarily given haloperidol injection 5 mg im st at 22:29 due to restlessness, running around, refusing to eat and take medication. Blood tests results included WBC, 14.09 × 10^9^/l, N, 83.3%, CK, 1016 U/L, and Myog, 30 ng/ml, and no other abnormalities. On the second day, she walked instability with muscle tone +  +  + . Considering that the drug caused the extrapyramidal system damage, she received scopolamine hydrobromide 0.3 mg im st and trihexyphenidyl 2 mg bid.

On the third day, the patient’s mental symptoms were prominent with muscle tone +  +  +  + and phlegm in the throat. Examination results showed that CK was 4444 U/L, AST was 128 U/L, NA + was133mmol/l, WBC was 12.75 × 10^9^/l, and N was 81.3%. She was diagnosed as atypical schizophrenia, extracortebral system reaction and secondary myolysis. Then the haloperidol was stopped and risperidone oral disintegrating tablets 2 mg qd and diazepam 10 mg q12h were given. On the 5th day, CK was 2406 U/L, AST was 114 U/L, NA + was 127.8 mmol/l, and the patient was in a rigid state. On the 8th day, the drug was adjusted to risperidone oral disintegrating tablets 3 mg qd and alprazolam 0.4 mg qn instead of diazepam. On the 14th day, she was fevered (T 39℃), and R was 30 times/min, P was 129 beats/min, BP was 128/80 mmHg. She also had dyspnea, phlegm in the throat, muscle tone +  +  +  + , tremor, and sweating. Her expression was dull, the consciousness was unclear, no definite dry and wet sounds found in both lungs, and the pathological symptoms were not elicited. Examination results were: WBC, 18.7 × 10^9^/l, N, 89.3%, CRP, 70.8 mg/l, PCT, 0.14 ng/ml, CK, 3639 U/L, Myog > 1000 ng/ml, NA + , 133.1.0 mmol/l, K + , 3.32 mmol/. Sputum culture result was haemophilus influenza. Arterial blood gas analysis demonstrated metabolic acidosis combined with metabolic alkalosis. Lung CT scan result suggested multiple exudative lesions in both lungs, which were mainly in the right upper lobe, middle and lateral lobe, lower lobe and left lower lobe. Then she was transferred to the respiratory department and was diagnosed as pneumonia, NMS and atypical schizophrenia. After MDT consultation, the risperidone was stopped and the patient was started on lorazepam 0.5 mg bid, levofloxacin 0.5 g qd anti-infection, and betaloc 12.5 mg bid. The airway management, hydration and other symptomatic supportive treatments were strengthened, and she was also received severe monitoring.

On the 16th day, the patient’s body temperature dropped to 38.0℃, R was 35 times/min, P was 148 beats/min, and BP was 154/92 mmHg. She had nervousness, constant groaning, tremor, stiffness, profuse sweating, phlegm in the throat, stomach retention, and constipation and no rales in both lungs. Arterial blood gas analysis showed decompensated metabolic acidosis combined with respiratory alkalosis. WBC was 9.97 × 10^9^/l, N was 83.9%, CRP was 58.31 mg/l, CK was 331 U/L, and Myog was 75 ng/ml. Considering that infection and myolysis have been corrected, but muscle tone and nervousness have not been improved, the treatment plan was then adjusted as: lorazepam 1.0 mg bid (taking in the morning and noon), 1.5 mg qn, betaloc 25 mg bid, and granaduonium choline hydrobromide 0.3 mg bid. Other treatments and monitoring measures were continued. On the 18th day, the patient’s vital signs were normal, tremor and throat sputum sound disappeared, muscle tone was +  +  + , and she was able to communicate but auditory hallucinations were obvious, and she cried and laughed sometimes. Routine biochemistry tests and arterial blood gas analysis results showed normal. Lung CT scan result revealed that the exudative lesion in the lung was completely absorbed. After consultation in the psychiatric department, she was given olanzapine 5 mg qn, and the anti-infection and hydration treatment were stopped. On the 21st day of admission, she developed fever again (T 38.0℃), R was 28 times/min, P was 130 beats/min, BP was 122/78 mmHg, the aforementioned symptoms were aggravated. Examinations showed that WBC was 8.7 × 10^9^/l, N was 77.2%, CRP was 31.8 mg /l, CK was 296 U/L, and Myog was 51 ng/ml. Thus, the olanzapine was stopped immediately and she was treated with fluid rehydration therapy. There was no fever on the 22nd day, and the auxiliary examination was normal. She was transferred to psychiatric department to receive MECT treatment. The detailed information about the laboratory tests was presented in Table 3.

**Table 3:** Changes in the laboratory tests in different days of Case 3

**Days after Admission**	**T (℃)**	**R(times/min)**	**P(beats/min)**	**BP (mmHg)**	**WBC (× 10**^**9**^**/l)**	**N (%)**	**CRP (mg/l)**	**PCT (mg/l)**	**Myog (ng/ml)**	**CK (U/L)**
0	36.5	20	80	110/70	14.09	83.3			30	1016
3					12.75	81.3				4444
5										2406
14	39	30	129	128/80	18.7	89.3	70.8	0.14	> 1000	3639
16	38	35	148	154/92	9.97	83.9	58.31		75	331
21	38	28	130	122/78	8.7	77.2	31.8		51	296

*Abbreviations*: *T* Body temperature, *R* Respiratory rate, *P* Pulse, *BP* Blood pressure, *WBC* White blood cell, *N* Neutrophil, *CRP* C-reactive protein, *PCT* Procalcitonin, *Myog* Myoglobin, *CK* Creatine kinase

## Discussion and conclusions

It is reported that the occurrence of NMS is related to antipsychotic drugs, antimania drugs, and some antidepressants [5]. It is also seen in the withdrawal or adjustment stage of Parkinson’s drugs [6]. The incidence of multi-drug combination is higher and high valence antipsychotics are higher than other dosage forms, such as haloperidol, etc. NMS is easily prone to pneumonia, rhabdomyolysis and other problems [7]. Currently, the clinical diagnosis is based on the diagnostic criteria of NMS in the American Psychiatric Association (DSM-V) [8]. NMS is rare in clinical practice, and there is little research on it. Its autonomic nerve dysfunction and systemic complications could directly lead to the deaths of patient with NMS. After more than 60 years of development, its mortality rate has declined. The first reported mortality rate for NMS patients in the 1960s was 76% and now the more recent mortality estimates range from 10 to 20% [9, 10]. The recommendations for NMS-specific drug treatment are based on case reports and clinical experience. However, there is no data support from clinical trials, and their effectiveness is unclear and controversial [11].

None of the complete data involved studies on complicated pneumonia or pulmonary infection. According to the review of domestic and foreign literatures, most of them focused on case reports, and researches related with combined pneumonia were rare. The 6 patients in the two literatures with relatively complete data [12, 13]. Among the 9 cases (6 cases in the literatures and 3 cases in our study), there were 3 males and 6 females, aged from 31 to 66 years old, (mean ± SD was 47.44 ± 12.34 years old). The time of NMS after the use of antipsychotics ranged from 2 days to 16 years (1 case was onset after 16 years of treatment), which was mainly concentrated within 1 week after taking antipsychotics. The diagnosis and severity assessment of 9 patients complied with pneumonia were all in accordance with relevant guidelines [14], and all were monitored and treated in a timely manner. Finally, 8 cases were cured, and 1 case was discharged automatically due to critical condition.

The commonly used drugs for NMS include dantrolene, bromocriptine and amantadine. It is indisputable that effective supportive treatment is indispensable for NMS [15]. However, it is observed that bromocriptine may prolong the course of the disease or lead to more sequelae. According to the availability of the drug and the marginal effect on the cardiovascular and respiratory system [16], 9 patients were all treated with diazepam drugs, supplemented with propranolol, scopolamine hydrobromide (or trihexyphenidyl) and hydration therapies. Only 2 cases were treated with bromocriptine. There are few clinical reports on NMS complicated with pneumonia, and the treatment experience is also limited. Nine cases were complicated with pneumonia and respiratory failure, and 8 cases of them were corrected by anti-infection, airway management, and oxygen therapy (including assisted breathing). All patients with NMS have problems such as autonomic nerve dysfunction, strong secretion of salivary glands, phlegm retention in the throat, throat muscle incoordination, difficulty in voluntary sputum and other problems, which can easily lead to aspiration pneumonia [14].

In conclusion, the use of diazepam and propranolol in the treatment of NMS is not conducive to sputum discharge and can aggravate respiratory failure. Therefore, oxygen therapy and airway management are very important. Take-home messages in this paper could be as follows. Firstly, NMS complicated with pneumonia requires early recognition, early diagnosis, early treatment, early MDT intervention, and timely admission to RICU. Secondly, timely discontinuation of antipsychotic drugs or re-use of the original antiparkinsonian drugs. Thirdly, in the condition of endotracheal intubation, it is necessary to use lorazepam and propranolol timely and in a sufficient amount. Fourth, hydration therapy (for myolysis patients), nutritional support, symptomatic treatment, and correction of electrolyte disorders are equally important. Fifth, use anti-infective treatment according to HAP guidelines [14], but avoid the adverse effects of antibiotics on neuromuscular and kidney. Finally, monitor inflammatory indicators, muscle enzymes, arterial blood gas analysis, coagulation function, ECG and non-invasive cardiac output, routine biochemical tests, skin humidity, heart rate, respiratory rate, muscle tone, etc., for daily assessment of the condition.

## Data Availability

Not applicable

## Abbreviations

NMS: Neuroleptic malignant syndrome
T: Body temperature
R: Breathing
P: Pulse
BP: Blood pressure
WBC: White blood cell
N: Neutrophil
CRP: C-reactive protein
SAA: Amyloid
PCT: Procalcitonin
Myog: Myoglobin
CK: Creatine kinase
FDP: Fibrinogen
Lac: Lactic acid
MDT: Multidisciplinary
SPO_2_: Saturation of haemoglobin with oxygen
CT: Aomputer tomography
AST: Aspartate transaminase
ALT: Alanine transaminase
